# Association of ATG16L1 gene haplotype with inflammatory bowel disease in Indians

**DOI:** 10.1371/journal.pone.0178291

**Published:** 2017-05-19

**Authors:** Srinivasan Pugazhendhi, Kirankumar Baskaran, Srikanth Santhanam, Balakrishnan S. Ramakrishna

**Affiliations:** Wellcome Trust Research Laboratory, Christian Medical College, Vellore, India; Indiana University School of Medicine, UNITED STATES

## Abstract

Inflammatory bowel disease (IBD) is characterized by multigenic inheritance. Defects in autophagy related genes are considered to show genetic heterogeneity between populations. We evaluated the association of several single nucleotide polymorphisms (SNPs) in the autophagy related 16 like 1 (*ATG16L1*) gene with IBD in Indians. The ATG16L1 gene was genotyped for ten different SNPs using DNA extracted from peripheral blood of 234 patients with Crohn’s disease (CD), 249 patients with ulcerative colitis (UC) and 393 healthy controls The SNPs rs2241880, rs4663396, rs3792106, rs10210302, rs3792109, rs2241877, rs6737398, rs11682898, rs4663402 and rs4663421 were genotyped using the Sequenom MassArray platform. PLINK was used for the association analysis and pairwise linkage disequilibrium (LD) values. Haplotype analysis was done using Haploview. All SNPs were in Hardy Weinberg equilibrium in cases and controls. The G allele at rs6737398 exhibited a protective association with both CD and UC. The T allele at rs4663402 and C allele at rs4663421 were positively associated with CD and UC. The T allele at rs2241877 exhibited protective association with UC only. The AA genotype at rs4663402 and the GG genotype at rs4663421 were protectively associated with both CD and UC. Haplotype analysis revealed that all the SNPs in tight LD (D’ = 0.76–1.0) and organized in a single haplotype block. Haplotype D was positively associated with IBD (P = 5.8 x 10^−6^ for CD and 0.002 for UC). SNPs in ATG16L1 were associated with IBD in Indian patients. The relevance to management of individual patients requires further study.

## Introduction

Inflammatory bowel disease (IBD), comprising Crohn’s disease (CD) and ulcerative colitis (UC), is considered to result from abnormal immune reactions to gut luminal microbiota in genetically predisposed individuals [1–3]. The genetic predisposition is contributed by multiple genes through common variants (single nucleotide polymorphisms or SNPs) that each appear to exert a small influence. The discovery that CD was associated with mutations in the *NOD2* gene [4,5] provided the first indication of the important role played by innate immunity in inflammation and disease progression. Subsequent genome wide association studies (GWAS) provided further insights into the genetic architecture of IBD, unravelling 163 susceptibility loci by the end of 2012 [3]. Disturbances in autophagy have been implicated as a potential pathogenetic pathway in IBD following the discovery that SNPs of genes involved in this pathway were associated with IBD [6].

Autophagy is a cellular process that involves the sequestration and eventual destruction of aging proteins, damaged cell organelles, apoptotic bodies and intracellular bacterial components by a specialized double membrane vesicle called the autophagosome. Autophagy plays a pivotal role in maintenance of immune homeostasis in the gut, contributing to both innate and adaptive immunity [7,8].

The link between autophagy and CD pathogenesis became first apparent when an association was identified between autophagy related 16-like 1 gene (*ATG16L1*) and CD [6]. *ATG16L1* encodes a small coiled coil protein which interacts with ATG5 and ATG12 to form a 350 kDa multimeric complex that plays a crucial role in the bulk degradation or autophagy of cytoplasmic proteins and organelles. ATG16L1 protein is expressed in the colon, small bowel, intestinal epithelial cells, leukocytes and spleen. A coding SNP, named rs2241880, in the *ATG16L1* was found to have a disease association with CD and this was responsible for threonine to alanine substitution (T300A) at amino acid 300 of protein. This SNP appeared to account for all of the disease risk exerted by the *ATG16L1* locus. Further replication studies in an independent UK cohort confirmed the association existing between autophagy and IBD, particularly CD [9,10]. However, the literature shows both presence of and lack of association of IBD with SNPs in the *ATG16L1* locus [11–16]. All of 9 *ATG16L1* SNPs that were genotyped in a German population displayed significant protective association with CD, the strongest association being with rs2241879 and rs2241880 [17]. Other *ATG16L1* gene variants independent of rs2241880 also appear to contribute to CD susceptibility [18]. Studies in several Asian countries including Japan, Korea and China failed to show an association between *ATG16L1* gene variants and CD [19–21].

In light of the conflicting findings and ethnic differences in aforementioned studies we therefore aimed at analyzing various polymorphisms in *ATG16L1* gene for its association with CD and UC in Indian population.

## Materials and methods

876 participants were recruited from patients attending the outpatient and inpatient services of the Department of Gastrointestinal Sciences at the Christian Medical College. The cohort comprised of 234 CD patients, 249 UC patients and 393 healthy controls (HC). The diagnosis of CD and UC was based on a composite of clinical, radiological, endoscopic, and histopathological findings according to the consensus criteria of the Indian Society of Gastroenterology [22,23]. Patients with proven intestinal or extra-intestinal tuberculosis were excluded. Participants who refused the consent to participate were also excluded. Healthy adults accompanying non-IBD patients to the Gastroenterology clinic were recruited as controls. The history and clinical details were recorded and samples of venous blood were obtained in EDTA-coated Vacutainer tubes.

### SNP genotyping

Genomic DNA was isolated from 8ml of EDTA–anticoagulated venous blood by salting out method. Isolated DNA was checked for quality and concentration, and stored at -80°C until analysis. Ten SNPs—rs2241880, rs4663396, rs3792106, rs10210302, rs3792109, rs2241877, rs6737398, rs11682898, rs4663402 and rs4663421 in *ATG16L1* gene were selected for genotyping. Our choice of SNPs for genotyping was based on the following considerations. The first four have been previously found associated with CD in German [6,17] and UK [24] patients, while the remaining six were selected from the Hapmap data of Gujarati Indians [25]. Of these SNPs, rs2241880 is a coding SNP, rs10210302 is located in the 5’ UTR region, and the remainder are located in intronic regions. In German patients, rs2241880 and rs2241879 have both been shown to be associated with CD, with the latter being in close LD with the former. In other studies, rs2241880 was the most replicated SNP, and we therefore chose to study rs2241880 but not rs2241879.

Genotyping of *ATG16L1* polymorphisms was performed using the Sequenom-MassArray platform at NxGenBio Life Sciences, New Delhi, India. As described earlier, in these assays a locus specific PCR reaction was carried out initially, followed by a locus specific primer extension (iPLEX) reaction in which an oligonucleotide primer annealed immediately upstream of the polymorphic site being genotyped. SNPs and small insertion/deletion polymorphisms were detected, by matrix-assisted laser desorption ionization–time-of-flight mass spectrometry after incubating primer and amplified DNA with mass-modified dideoxynucleotide terminators [26]. The primer extension was made according to the sequence of the variant site, and was a single complementary mass-modified base. Genotypes were assigned at each SNP locus using SpectroTYPER software (Sequenom).

### Statistical analysis

To determine association of *ATG16L1* SNPs with IBD susceptibility, comparison of allele and genotype distributions among cases and controls was done using PLINK v. 1.07 (website: http://zzz.bwh.harvard.edu/plink/) [27]. Odds ratios (ORs), 95% confidence intervals (CIs) and P-values were calculated. LD pairwise values, haplotype structure, and haplotype frequencies were determined using the Haploview software v. 4.2 [28]. Significance of difference between groups was analysed using Chi square test.

### Ethical considerations

The Institutional Review Board of the Christian Medical College approved the study protocol and consent forms. Informed written consent for genetic testing was obtained from all study participants and from parents of minors participating in the study.

## Results

Of the CD patients 152 were male and 82 female and their ages ranged from 10–78 years (median 44). Eighty eight had ileocolonic disease, 78 had ileal disease, 56 had colonic disease, 7 had upper GI involvement alone, and 5 had ileocolonic disease with involvement of upper GI tract. Forty three had stricturing disease, 22 had penetrating disease and the remaining had non-stricturing non-penetrating disease. Of the UC patients 145 were male and 104 female, and their ages ranged from 15 to 76 years (median 39). Thirty four had proctitis, 63 had left sided colitis and 152 had extensive colitis or pancolitis. The healthy controls, 256 male and 143 female, ranged in age from 15–73 years (median 30).

### SNP analysis

All investigated polymorphisms were in Hardy Weinberg equilibrium in cases and controls. The allele and genotype frequencies of *ATG16L1* polymorphisms are presented in Tables 1 and 2 respectively (see S1 Table for individual level data). Association analysis was carried out by comparing the allele and genotype frequencies of *ATG16L1* markers amidst cases and controls.

**Table 1:** Allele frequencies of ATG16L1 SNPs in cases and controls in Indian population.

Marker	Minor Allele	Controls MAF	Crohn’s disease			Ulcerative colitis		
			MAF	P-value	OR(95%CI)	MAF	P-value	OR(95%CI)
rs10210302	C	0.5118	0.4848	0.359	0.89(0.71–1.13)	0.4676	0.125	0.83(0.66–1.05)
rs6737398	G	0.4081	0.3398	0.017	0.74(0.58–0.94)	0.3525	0.048	0.78(0.62–0.99)
rs11682898	T	0.1671	0.1422	0.246	0.82(0.59–1.14)	0.1701	0.890	1.02(0.75–1.38)
rs2241880	T	0.514	0.5022	0.691	0.95(0.75–1.20)	0.4689	0.125	0.83(0.66–1.05)
rs3792109	C	0.5039	0.4806	0.428	0.91(0.72–1.14)	0.4694	0.232	0.87(0.69–1.09)
rs2241877	T	0.2428	0.2022	0.100	0.79(0.59–1.04)	0.1824	0.011	0.69(0.52–0.92)
rs3792106	A	0.3346	0.337	0.933	1.01(0.79–1.29)	0.3012	0.217	0.85(0.67–1.09)
rs4663396	T	0.2742	0.2851	0.679	1.05(0.81–1.36)	0.302	0.285	1.14(0.89–1.47)
rs4663402	T	0.0362	0.0909	5.9x10^-5^	2.65(1.62–4.35)	0.076	0.001	2.19(1.32–3.62)
rs4663421	C	0.0327	0.0968	2.4x10^-6^	3.17(1.91–5.25)	0.079	0.0002	2.55(1.52–4.28)

MAF = minor allele frequency. OR = Odds ratio. CI = Confidence interval.

**Table 2:** Genotype distributions of ATG16L1 SNPs in cases and controls.

SNP	Genotype	Controls (%)	Crohn’s Disease		Ulcerative Colitis	
			Patients	P-Value	Patients	P-Value
rs10210302	TT	89 (23)	49 (21.3)	0.04	59 (24)	0.04
	TC	194 (51)	139 (60.4)		145 (59)	
	CC	98 (26)	42 (18.3)		43(17)	
rs6737398	AA	137 (36)	98(42.4)	0.03	100(41)	0.09
	AG	177 (46)	109 (47.2)		116 (48)	
	GG	67 (18)	24 (10.4)		28 (11)	
rs11682898	CC	266 (69)	169(73)	0.41	165(68)	0.65
	CT	106 (28)	60 (26)		75 (30)	
	TT	11 (3)	3 (1)		4 (2)	
rs2241880	CC	84 (24)	47(20)	0.11	64(27)	0.22
	CT	178 (50)	135 (59)		128 (53)	
	TT	94 (26)	48 (21)		49 (20)	
rs3792109	TT	94(25)	54(23.3)	0.13	63(26)	0.22
	TC	189 (50)	133 (57.3)		134 (55)	
	CC	97 (25)	45 (19.4)		48 (19)	
rs2241877	CC	221(58)	144(63)	0.13	163(67)	0.04
	CT	135 (35)	79 (34)		73 (30)	
	TT	25 (7)	7 (3)		8 (3)	
rs3792106	GG	171(45)	95(41)	0.20	119(49)	0.44
	GA	165 (43)	115 (50)		103 (42)	
	AA	45 (12)	20 (9)		22 (9)	
rs4663396	CC	199(52)	111 (48.6)	0.54	110(45)	0.10
	CT	158 (41)	104 (45.6)		122 (50)	
	TT	26 (7)	13 (5.7)		13 (5)	
rs4663402	AA	358 (93)	190(82.3)	0.0001	212(85.1)	0.0029
	AT	28 (7)	40 (17.3)		36 (14.5)	
	TT	0	1 (0.4)		1 (0.4)	
rs4663421	GG	357(93)	188(81)	0.0001	208(85)	0.0006
	GC	25 (7)	43 (19)		35 (14)	
	CC	0	1		2 (1)	

#### rs4663402 and rs4663421

rs4663402 and rs4663421 exhibited unequivocal association with CD and UC in terms of both allele and genotype frequencies. The T allele of rs4663402 and the C allele of rs4663421 were associated with both CD (OR 2.65, 95% CI 1.62–4.35 and OR 3.17, 95% 1.91–5.25 respectively) and UC (OR 2.19, 95% CI 1.32–3.62 and OR 2.55, 95% CI 1.52–4.28 respectively) (Table 1). The genotype frequencies at rs4663402 and rs4663421 were also significantly associated with IBD (Table 2).

#### rs6737398

The minor G allele at this locus was protectively associated with IBD, its frequency being higher in controls compared to cases. Corresponding OR (95% CI) values were 0.74 (0.58–0.94) for CD and 0.78 (0.62–0.99) for UC (Table 1). The GG genotype at this locus was noted in 18% of HC compared to 10.4% of CD patients (Table 2).

#### rs10210302

Although the minor C allele of rs10210302 did not reveal significant association with IBD, the CC genotype of rs10210302 was protectively associated with IBD being present in 26% of HC compared to 18% of CD patients and 17% of UC patients (Table 2).

#### rs2241877

The minor T allele at the rs2241877 locus demonstrated a protective association with UC, but not CD (Table 1). Correspondingly, the TT genotype was protectively associated with UC (Table 2).

The allele and genotype frequencies of rest of the *ATG16L1* SNPs did not show a significant difference between patients and healthy individuals.

### Haplotype analysis

**Fig 1:**
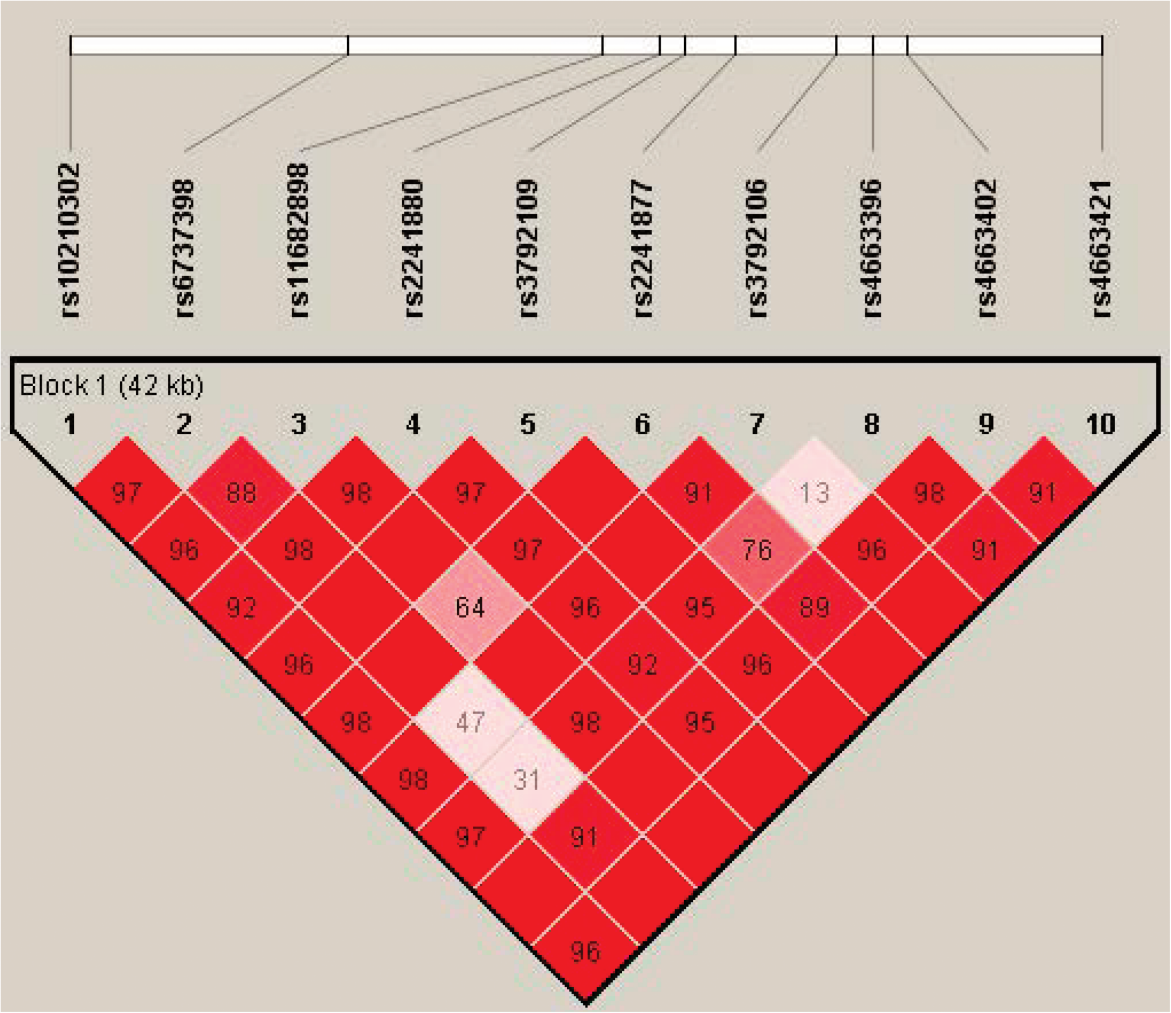
Linkage disequilibrium (LD) pattern between the ten *ATG16L1* single nucleotide polymorphisms (SNPs) genotyped in this study. All SNPs were in tight LD (D’ = 0.76–1.0) and were organized in a single haplotype block.

Nine haplotypes (A-I) were constructed from the ten *ATG16L1* SNPs (Table 3). Despite the existence of weak LD between SNPs rs3792106 and rs4663396 (D’ = 0.13), rs6737398 and rs4663396 (D’ = 0.31), rs6737398 and rs3792106 (D’ = 0.47) and rs11682898 & rs2241877 (D’ = 0.64), all the ten SNPs were organized in a single haplotype block (D’ ranging from 0.76–1.0) (Fig 1). On assessing the differences in haplotype frequencies amidst cases and controls it was found that haplotype D was positively associated with IBD being found in 8% of CD patients and 6% of UC patients compared to 2% of controls (P = 5.8x10^-6^ and 0.002 respectively for CD and UC). Haplotype B exhibited a protective association with UC being present in 16% of UC patients and 22% of HC (Table 4).

**Table 3:** Haplotype structure of ten *ATG16L1* SNPs.

Haplotype	rs10210302	rs6737398	rs11682898	rs2241880	rs3792109	rs2241877	rs3792106	rs4663396	rs4663402	rs4663421
A	T	A	C	C	T	C	G	C	A	G
B	C	G	C	T	C	T	A	C	A	G
C	C	G	T	T	C	C	G	T	A	G
D	C	A	C	T	C	C	A	T	T	C
E	C	A	C	T	C	C	A	T	A	G
F	C	G	C	T	C	C	A	T	A	G
G	T	A	C	T	T	C	G	C	A	G
H	C	G	T	T	C	T	G	T	A	G
I	C	A	T	T	C	C	G	T	A	G

**Table 4:** Haplotype frequency in healthy controls and IBD patients.

Haplotype	Control	CD	P-Value	UC	P-Value
A	0.47	0.48	0.63	0.51	0.15
B	0.22	0.17	0.06	0.16	0.01
C	0.14	0.11	0.24	0.15	0.72
D	0.02	0.08	5.8x10^-6^	0.06	0.002
E	0.04	0.03	0.56	0.02	0.28
F	0.02	0.01	0.37	0.01	0.33
G	0.02	0.01	0.99	0.01	0.48
H	0.01	0.01	0.70	0.008	0.70
I	0.01	0.006	0.18	0.006	0.16

## Discussion

In the present study we explored the association of IBD with a number of genetic variants in the *ATG16L1* gene. The SNPs rs4663421 and rs4663402 revealed a positive association with IBD. The SNPs rs6737398 and rs10210302 had significant protective associations, at the genotype level, with CD and UC. The protective association of rs2241877 was noted with UC alone. While specific alleles were associated, it became clear that a single haplotype was responsible for the strong association with both CD and UC.

Following the identification of genetic alterations within *ATG16L1* to be associated with CD and UC development by two genome wide association studies [6,24], a significant number of studies have replicated this association in populations of Caucasian origin [11,17], corroborating the relevance of *ATG16L1* to IBD genesis. Earlier data suggested that all the CD risk associated with *ATG16L1* could be fully attributed to the T300A variant [6]. Hence most studies confined themselves to analyzing the association of this marker with IBD. Notable exceptions were studies in the German [17] and Japanese [19] populations in which 9 and 12 SNPs respectively were typed. Strong association of T300A (rs2241880) with CD has been established in populations in New Zealand [11] [P = 0.0001, OR = 1.41, 95% CI = 1.18–1.67], Australia [16] [P< 0.001, OR = 1.96, 95% CI = 1.49–2.58], Netherlands [12] [OR = 1.36, 95% CI = 1.21–1.66, P = 0.0017], and Hungary [29] [P = 0.004, OR = 1.69, 95% CI = 1.19–2.41]. On the other hand, a lack of association between rs2241880 and CD has been reported from Italy [30], Lithuania [31] and Brazil [32]. Protective and positive associations of the T300A variant with UC have been reported in Australian [16] and UK [9] studies respectively. However, several other studies failed to find a significant association of T300A with UC [6,10]. In the present study rs2241880 did not have any significant association with either CD or UC. This finding is consistent with a study reported in the abstract in which 216 Indian Asian IBD patients (60 CD: 156 UC) were compared with 203 healthy Indian Asian controls and no association was found between rs2241880 and IBD [33].

*ATG16L1* is a member of a large family of genes involved in autophagy, a housekeeping cellular process by which degraded intracellular components, and excess and damaged organelles are digested and removed [34]. Autophagy is believed to have a role in immune defense against intracellular pathogens [35], which may explain why defects in this pathway are important in IBD pathogenesis. For instance, functional knockdown of the *ATG16L1* gene abrogates the autophagy of *Salmonella* Typhimurium. The T300A variant at rs2241880 in *ATG16L1* gene has been considered to be the key variant associated with IBD [6]. In a human intestinal epithelial cell line, defective antibacterial autophagy was noted in cells expressing the CD associated variant protein (Ala) compared to cells expressing the wild type protein (Thr) [36], demonstrating that the non-synonymous SNP is associated with altered responses to bacterial components. In a study of ileal tissue associated microbiota from patients with ileal CD, patients homozygous for the *ATG16L1* risk allele had abundant Fusobacteriaceae, whereas patients homozygous for the *ATG16L1* protective allele showed reduced abundance of Bacteroidaceae and Enterobacteriaceae [37]. Monocytes from these patients showed impaired killing of adherent invasive *Escherichia coli* in patients homozygous for the ATG16L1 risk allele compared with those homozygous for the protective allele [37].

The SNPs rs3792106 and rs4663396 were associated protectively with CD (OR 0.77, 95% CI 0.67–0.87 and OR 0.81, 95% CI 0.68–0.95 respectively) in a German population [17]. On the other hand, these SNPs did not show any association with CD in Japanese [19]. In the present study, no significant association was detected between these SNPs and CD.

The SNP rs10210302 was very significantly associated with CD in the Wellcome Trust Case Control Consortium study [34]. However, a study in UC patients failed to identify any significant association between rs10210302 and UC [38]. In the present study the homozygous CC genotype of rs10210302 was significantly protectively associated with both CD and UC. It should be noted that the rs10210302 polymorphism was not associated with CD in an Ashkenazi Jewish cohort [39]. A meta-analysis (including 15 studies with 9211 UC and 10899 controls) of the association between UC and rs2241880 indicated a significant association between the G allele and increased risk for UC (odds ratio 1.08, p = 0.0003) [40]. The above comparisons indicate that there is significant variation with regard to the frequencies of *ATG16L1* variants, and their association with IBD, in ethnically divergent populations. A recent meta-analysis concluded that *ATG16L1* was not associated with IBD in East Asians [41]. However an analysis of IBD genetics data from 86,640 European individuals and 9,846 individuals of East Asian, Indian or Iranian descent concluded that heterogeneity between populations in IBD genetics was driven, in the case of ATG16L1, by effect size rather than by allele frequency [42].

Of interest, the present study also revealed significant associations between SNPs rs4663421, rs4663402, rs6737398 and rs2241877 in the *ATG16L1* gene and IBD. There is as yet no data on the association of these variants with IBD in other populations.

It is also to be noted that, with the exception of a single Japanese study [19], haplotype analysis has not been done with the SNPs of *ATG16L1* in any of the association studies in various populations. Of the nine haplotypes (A-I) that we identified in our population, only one was majorly associated with both forms of IBD.

The functional consequence of these mutations can only be indirectly inferred as there are no studies which directly examine the influence of these SNPs on ATG16L1 expression. In the present study the coding SNP rs2241880 was not associated with IBD in the population studied. However SNPs rs4663421 and rs4663402 were significantly associated with IBD and were included in the haplotype that was associated with IBD. Using HaploReg (http://archive.broadinstitute.org/mammals/haploreg/haploreg_v3.php) we identified that these two SNPs are in close linkage disequilibrium (0.97 for rs4663421 and 0.91 for rs4663402) with rs140725980 (S2 Table). The latter SNP (rs140725980) is an enhancer histone mark for ATG16L1 gene in gastrointestinal tissue. Enhancer histone marks flank sites of transcription factor binding and enhancer activity and histone marked active enhancers are associated with level of gene expression. Methylation of histone H3K4 is tightly associated with the promoters of active genes. Thus it is possible that the haplotype that we identified is associated with altered expression of ATG16L1 in gastrointestinal tissues, explaining a role for the haplotype in the genesis of IBD.

This is the first study showing significant association of *ATG16L1* variants rs4663421, rs4663402, rs6737398 and rs2241877 with CD and UC in Indian population, identifying autophagy as a host pathogen defense pathway crucial to IBD biology. Further elucidation of role of this process in IBD pathogenesis will be facilitated by detailed investigation into the functional consequences of these variants, besides exploring other proteins involved in autophagy pathway.

## Supporting information

S1 Table This table shows the individual level data on each allele in each participant. Following are the expansions and codes: PID is the participant ID. Sex, 1 = male, 2 = female. Diagnosis, 1 = control, 2 = Crohn’s disease, 3 = ulcerative colitis. For individual SNPs each allele is shown separately, 1 = A, 2 = C, 3 = G, 4 = T, and 0 = untypable.
(XLSX)

S2 Table This table shows the HaploReg output when queried for the associations of rs4663402 and rs4663421. (XLSX)
